# The Disordered EZH2 Loop: Atomic Level Characterization by ^1^H^N^- and ^1^H^α^-Detected NMR Approaches, Interaction with the Long Noncoding HOTAIR RNA

**DOI:** 10.3390/ijms23116150

**Published:** 2022-05-30

**Authors:** Csenge Lilla Szabó, Beáta Szabó, Fanni Sebák, Wolfgang Bermel, Agnes Tantos, Andrea Bodor

**Affiliations:** 1 Analytical and BioNMR Laboratory, Institute of Chemistry, Eötvös Loránd University, Pázmány Péter Sétány 1/A, 1117 Budapest, Hungary; lilla.szabo@ttk.elte.hu (C.L.S.); fanni.sebak@ttk.elte.hu (F.S.); 2 Hevesy György Ph.D. School of Chemistry, Eötvös Loránd University, Pázmány Péter Sétány 1/A, 1117 Budapest, Hungary; 3 Institute of Enzymology, Research Centre for Natural Sciences, Magyar Tudósok Körútja 2, 1117 Budapest, Hungary; szabo.beata@ttk.hu; 4 Bruker BioSpin GmbH, Rudolf-Plank Str. 23, 76275 Ettlingen, Germany; wolfgang.bermel@bruker.com

**Keywords:** EZH2, intrinsically disordered proteins, IDP–RNA interaction, HOTAIR, fuzzy complex, ^1^H^α^ detected NMR

## Abstract

The 96-residue-long loop of EZH2 is proposed to play a role in the interaction with long non-coding RNAs (lncRNAs) and to contribute to EZH2 recruitment to the chromatin. However, molecular details of RNA recognition have not been described so far. Cellular studies have suggested that phosphorylation of the Thr345 residue localized in this loop influences RNA binding; however, no mechanistic explanation has been offered. To address these issues, a systematic NMR study was performed. As the ^1^H^N^-detected NMR approach presents many challenges under physiological conditions, our earlier developed, as well as improved, ^1^H^α^-detected experiments were used. As a result of the successful resonance assignment, the obtained chemical shift values indicate the highly disordered nature of the EZH2 loop, with some nascent helical tendency in the Ser407–Ser412 region. Further investigations conducted on the phosphomimetic mutant EZH2^T345D^ showed that the mutation has only a local effect, and that the loop remains disordered. On the other hand, the mutation influences the *cis/trans* Pro346 equilibrium. Interactions of both the wild-type and the phosphomimetic mutant with the lncRNA HOTAIR_140_ (1–140 nt) highlight that the Thr367–Ser375 region is affected. This segment does not resemble any of the previously reported RNA-binding motifs, therefore the identified binding region is unique. As no structural changes occur in the EZH2 loop upon RNA binding, we can consider the protein–RNA interaction as a “fuzzy” complex.

## 1. Introduction

Epigenetic regulator proteins play key roles in cell proliferation and differentiation. The enhancer of zeste homologue 2 (EZH2), a part of the gene-regulating polycomb repressive complex 2 (PRC2), is responsible for the trimethylation of histone H3 lysine 27 (H3K27), resulting in the silencing of several genes, including tumor suppressors. This effectively fuels cancer proliferation [1]. Recent scientific evidence has highlighted the role of long non-coding RNAs (lncRNAs) in the targeting of the PRC2 complex [2,3]. Amongst the PRC2 subunits, EZH2 shows the highest affinity towards lncRNAs [4,5], although it lacks a well-defined RNA-binding motif [6]. A great variability was observed among the binding partners [7,8], and EZH2 was postulated to be promiscuous for RNAs [9], albeit it can discriminate between different lncRNAs, and its specificity can be fine-tuned in vivo by other factors [4,5,10]. Details of the RNA features that the EZH2 can recognize remain elusive, as many but not all of the interacting RNA partners are reported to fold into secondary structural elements [11,12]. This includes the important binding partner, *HOX* Transcript Antisense Intergenic RNA (HOTAIR) [13]. This lncRNA plays a crucial role in the recruitment of the PRC2 complex to its target genes [5,14,15]. Two binding motifs, a duplex and a quadruplex structure, were proposed [5], and recent studies have suggested that the G-quadruplex is more likely to interact with the PRC2 [16,17].

**Figure 1:**
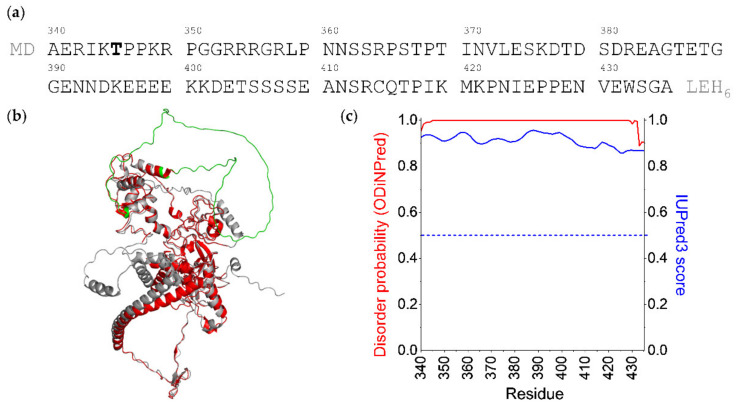
Characteristics of the studied EZH2 segment: (**a**) Amino acid sequence of the EZH2 loop region (UniProt Q15910), highlighting the mutation site Thr345; N-terminal MD and C-terminal LEH_6_ residues are cloning artifacts. (**b**) Crystal structure (red) [6] of EZH2 superimposed with the AlphaFold predicted structure (gray). The studied loop region is highlighted with green. (**c**) Disorder probability predicted with ODiNPred [24] (red) and IUPred3 [23] scores (blue). The IUPred score indicates a disordered nature over 0.5.

Since EZH2 does not contain a canonical RNA-binding element, the location of the RNA-binding site on EZH2 is debated in the literature. Long et al. reported on different key binding residues throughout EZH2, most of which are located in the N-terminal helical region, and they noted that residues from other PRC2 components may be involved in RNA binding as well [18]. Based on literature information, it appears that RNA binding is mediated by a fragmented binding surface formed by different PRC2 subunits and several RNA-recognizing regions within separate proteins [4]. One of the key questions that remains still unanswered is the regulation and specificity of the PRC2-RNA interaction, as PRC2 shows promiscuous RNA binding in vitro [9], yet elicits highly specific reactions to certain RNAs in vivo [19]. An indication of in vivo regulation emerged when it was shown that RNA binding and subsequent PRC2 recruitment is enhanced upon the phosphorylation of Thr345 [13,20]. The reported region, including Thr345, is located at the beginning of a 96-residue-long loop of EZH2 (Figure 1a), which is considered to be disordered, as suggested by the missing electron densities from the X-ray structure of PRC2 [6]. Its presumed disordered nature was further confirmed by the AlphaFold-generated structure [21,22], in which the per-residue confidence score is below 50 for the whole loop region (Figure 1b). Moreover, the disorder prediction programs IUPred3 [23] and ODinPred [24] show a high propensity of the sequence to be unstructured (Figure 1c). Structural effects and the mechanistic background of the effect of phosphorylation has not been offered, and no explanation is given as to how phosphorylation might increase affinity to RNA molecules.

RNA-binding intrinsically disordered protein regions (IDRs) have received increasing attention during the previous years, as more and more information surfaces on their abundance and biological relevance [25,26]. While there are well-known disordered RNA-recognition elements, such as the RGG motifs or the serine-rich regions, many identified RNA-binding disordered segments lack these signature characteristics [27]. Scarcity of detailed structural studies of these complexes render the molecular details of the RNA recognition by these IDRs still enigmatic. A closer inspection of the EZH2 loop sequence (Figure 1a) reveals that it does not resemble neither the RGG nor the serine-rich type RNA-binding IDRs. Thus, studying its RNA-binding capabilities not only offers a unique opportunity to understand how a non-canonical, RNA-binding IDR recognizes its cognate partner, but also helps us uncover the structural background of the phosphorylation that may fine-tune protein–RNA recognition.

Since disordered proteins are famously recalcitrant to crystallize, NMR spectroscopy is the only method [28] to give an atomic-level characterization. Nevertheless, physiological conditions (pH~7.0, 310 K) may pose challenges for the well-known ^1^H^N^-detected approach. Proline residues, which are more abundant in IDPs than in folded proteins [29], and lack H^N^ protons, further complicate assignment strategies, especially if Pro–Pro motifs are present. Moreover, proline *cis/trans* isomerization might give rise to minor components, and a certain equilibrium is established [30].

In the light of all these, we aimed to understand how the disordered loop of EZH2 recognizes one of its most important RNA partners, HOTAIR, and whether phosphorylation modifies the interaction. To achieve these goals, we performed a detailed atomic-level characterization, and we studied the changes caused by the phosphomimetic mutation and characterized the interaction with the HOTAIR_140_ (1–140 nt) lncRNA, localizing binding regions on the EZH2 loop.

## 2. Results

### 2.1. Comparison of ^1^H^N^ and ^1^H^α^ Detected Approaches

**Figure 2:**
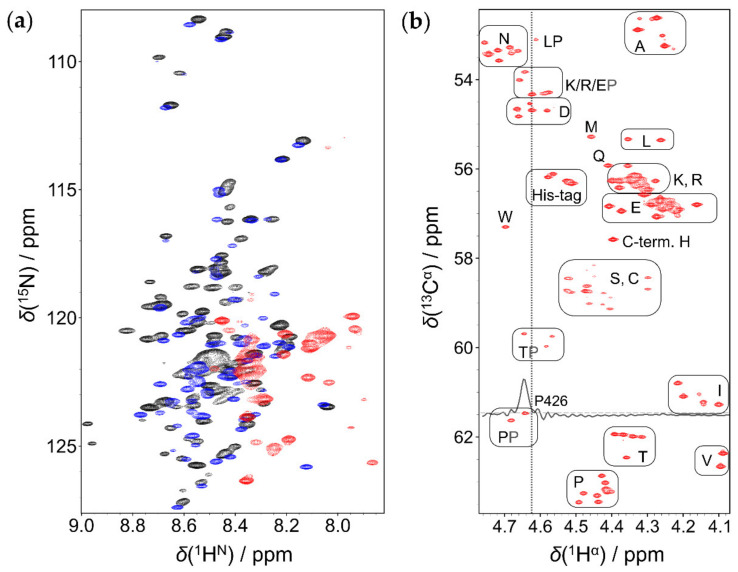
Comparison of ^1^H^N^ and ^1^H^α^ detected methods: (**a**) ^1^H,^15^N-HSQC at 310 K, pH = 7.2 (red), at 278 K, pH 7.2 (blue), and 278 K, pH = 5.1 (black). (**b**) SHACA-HSQC at 310 K, pH = 7.2. Amino acid types are shown with boxes (except glycines), the residual water signal is indicated with a light grey dashed line at 4.62 ppm. 1D row at *δ*_Cα_ = 61.47 ppm is shown to illustrate sensitivity for Pro426 in the proximity of the residual water signal.

Under physiological conditions, the performance of the classical ^1^H^N^ detected method is limited due to the fast chemical exchange of amide protons with water [31]. For the EZH2 loop, the ^1^H,^15^N-HSQC measurement at 310 K, pH = 7.2 delivered only ~30% of the expected signals (Figure 2a) due to broadening of several resonances below the detection limit. The problem was solved when working at a lower temperature (278 K), the assignment of ~85% of the peaks was already done. Acidic pH helped further: at pH = 5.1, a 92% assignment was achieved.

However, EZH2 interaction with HOTAIR_140_ requires conditions close to physiological, while, at the NMR-optimized conditions of low temperature and acidic pH, it does not take place. ^1^H^α^-detected methods represent a possibility to achieve peak assignment under these circumstances. H^α^ protons are not affected by chemical exchange and each proteinogenic amino acid, including proline, possesses them. Our earlier introduced SHACA-HSQC correlation experiment [32], including the real-time homo- and heteronuclear decoupled detection scheme, BASEREX [33], has an outstanding resolution, and most signals are well resolved (Figure 2b). Peaks close to the remaining water signal (*δ* = 4.62 ppm at 310 K) are also clearly visible, as shown in Figure 2b for the 1D row of Pro426. A nice clustering of the different amino acid types can be observed. Note that in the glycine region, line broadening is due to the geminal ^1^H-^1^H couplings (Figure S1).

To perform sequential assignment, the already reported ^1^H^α^-detected experiments [34,35] were used at 278 K and physiological pH. Under these conditions, the water signal is detected at 5.02 ppm, causing no disturbance in the ^1^H^α^ region (4.8 ppm–4.0 ppm). Amide ^15^N and carbonyl ^13^C chemical shifts can be obtained from the 3D HCAN, its complementary 3D HCA(CO)N, as well as from the 3D HCA(N)CO experiments. The information gained from both ^1^H^N^- and ^1^H^α^-detected approaches under these conditions result in a nearly complete backbone assignment, including 100% of ^13^C^α^ and ^15^N chemical shifts, with some ^1^H^N^, ^1^H^α^, and carbonyl ^13^C missing due to severe signal overlap. As, at low temperature, the water peak is positioned favorably downfield from the ^1^H^α^ region, the classical measurements can be used without further modification. However, at 310 K, the water peak situated at 4.62 ppm is overlapping with the ^1^H^α^ region, and a loss of ~15% of signals in the 3D experiments is observed. To overcome this effect, improvements are needed. Solutions to obtain a more efficient water peak removal and a better phase behavior are: the addition of a weak presaturation, a pair of low power 90° pulses with phases x, y before the composite pulse decoupling (CPD) block on ^1^H [36], a purge block [37], and the inclusion of the BASEREX detection scheme [33], as well as additional gradients during the back–INEPT transfer (Figure S2a,b). All these additions result in a remarkable improvement for peak assignment. For example, in case of Thr367, Pro426, and Glu425, for which highly distorted or no peaks could be observed using the classical pulse sequence (Figure S2c), the improved sequence allows clearly detectable signals (Figure S2d). Only Asn360 and 4 Asp residues in the proximity of the water signal remained undetectable. Assignments at both 278 K and 310 K were deposited in the Biological Magnetic Resonance Bank (BMRB, Entry 51420).

### 2.2. Secondary Structural Propensities

The signal dispersion of both ^1^H^N^ and ^1^H^α^ dimensions is only ca. 1 ppm (Figure 2a,b), indicating a similar chemical environment for each residue (Figure 2b). This is already indicative of a protein without any defined structure. To gain a better insight into the structural propensities, we analyzed the secondary chemical shifts (SCS) of ^13^C^α^ atoms. To do so, random coil (RC) chemical shifts were determined using two methods that consider the effect of both temperature and pH. The predictor developed by Kjaergaard et al. uses experimentally determined chemical shifts of short peptides [38,39], while the POTENCI uses a deep learning algorithm trained on a large dataset of IDPs and IDRs [40]. As the chemical shifts of IDPs are always close to the RC values, we believe that comparing the result of two RC predictors based on different algorithms will provide a better insight about the structural propensities.

**Figure 3:**
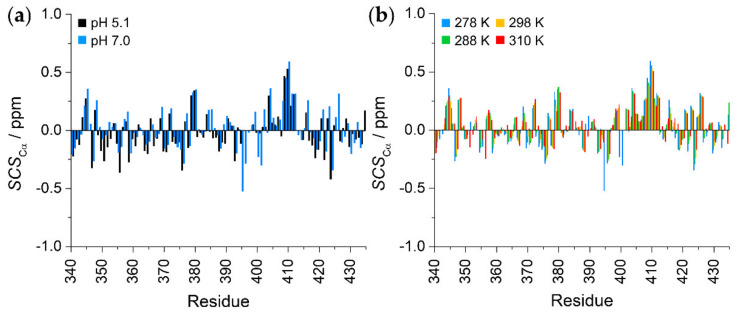
^13^C^α^ secondary chemical shifts of the EZH2 loop: (**a**) pH dependence of SCS values at 278 K. (**b**) Temperature dependence of SCS values at pH = 7.2. Random coil chemical shifts were calculated with POTENCI [40].

Results show that the two predictors give a similar picture (Figure 3 and Figure S3a,b), with SCS values close to 0.0, and no expanded constant sign tendencies are observed. The only exception is the Ser407–Ser412 segment with a helical tendency, although, even in this case, SCS values are below 0.6 ppm. This suggests a highly disordered random coil nature with a short nascent helical motif at Ser407–Ser412. Furthermore, we can observe that the secondary structure propensities do not depend on pH (Figure 3a) or on temperature (Figure 3b). This observation is somewhat contradictory, with the expectation being that, at a lower temperature, a more rigid transient structural tendency is obtained [41]. On the other hand, this indicates that the EZH2 loop is highly flexible under all studied conditions. The Secondary Structural Propensity (SSP) [42] calculator also confirms these findings (Figure S3c). Temperature coefficients of amide protons indicate that they are not involved in hydrogen bonds [43], further supporting the disordered nature of the EZH2 loop (Figure S3d).

### 2.3. The Effect of T8D Mutation and Proline Isomers

**Figure 4:**
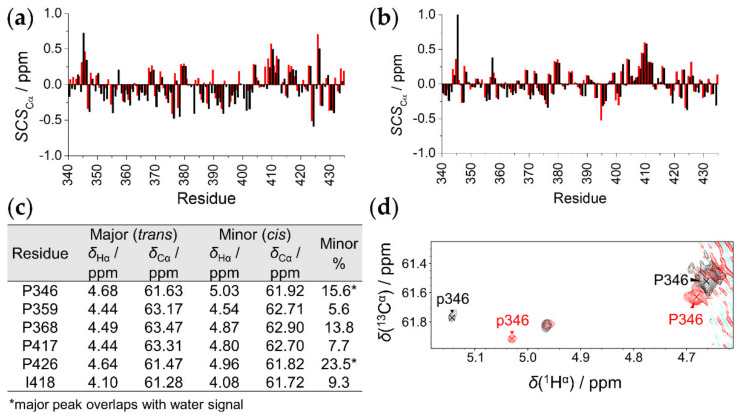
Comparison of EZH2^wt^ and EZH2^T345D^: (**a**,**b**) SCS_Cα_ values of EZH2^wt^ (red) and EZH2^T345D^ (black). Random coil values were calculated according to Kjaergaard et al. (**a**) and with POTENCI (**b**). (**c**) Determined *cis*/*trans* Pro conformers with chemical shifts and percentage of minor conformer indicated. (**d**) Proline region of the SHACA-HSQC spectrum of EZH2^wt^ (red) and T345D mutant (black) at 310 K. p346 refers to the minor signal of P346.

It was previously documented that phosphorylation of Thr345 increases affinity towards RNA [13]. As a first step, to understand the structural background of this effect, we studied the EZH2^T345D^ phosphomimetic mutant. This substitution introduces a negative charge, mimicking the Thr345-phosphorylated protein. Investigations for EZH2^T345D^ were performed at 278 K and 310 K, using both ^1^H^N^- and ^1^H^α^-detected assignment strategies. Comparison shows that ^15^N, ^1^H^N^, and ^13^C^α^ chemical shift differences between the wild type and T345D mutant are insignificant. The exception is the region in the vicinity of the mutation; the backbone chemical shifts of Pro346 suffer the highest deviations (BMRB Entry 51421). SCS values of ^13^C^α^ atoms calculated with the predictor of Kjaergaard et al. [38,39] and POTENCI [40] (Figure 4a,b) are below 1 ppm with similar tendencies for the two variants. This suggests that the T345D mutant is highly disordered, with similar secondary structural propensities as the wild type. The mutation affects only the chemical environments of the neighboring residues and has no global effect on the IDP.

Regarding the proline environments in the protein, we already showed that, in IDPs, a certain *cis/trans* proline equilibrium is established [44]. Indeed, minor peaks are detected in the SHACA-HSQC spectrum for the wild-type protein. Proline selective measurements [30] revealed chemical shift values for the ^13^C^β^ and ^13^C^γ^ environments that are indicative of the isomeric form. All detected minors show the *cis* conformer, while, in the major form, the expected *trans* conformer is present (Figure S4). The intensities of minor peaks were 5–15% relative to the sum of the minor and the corresponding major peak intensities (Figure 4c). Minor prolines are also detected for the EZH2^T345D^ mutant (Figure 4d). A closer analysis shows that, while for the major, *trans* Pro346 deviation is small, 0.02 ppm in ^1^H^α^; in case of the minor *cis* form, the chemical shift perturbance is more significant, with a ^1^H^α^ shift of 0.12 ppm. This alteration suggests that the phosphorylated EZH2 adopts a more turn-like structure at Pro346 in its minor conformer, which may affect the biological function of the IDP.

### 2.4. Interaction of HOTAIR_140_ with the EZH2 Loop

HOTAIR_140_ is prone to form a G-quadruplex structure, and it is reported to bind EZH2. A qualitative analysis based on the inspection of ^1^H 1D spectra can reveal the existence of the quadruplex structure, which is promoted by Mg^2+^ cations. Even though the studied RNA segment is long, and only broad signals can be detected, we compared the ^1^H 1D spectra in the absence and presence of magnesium salts. In the absence of Mg^2+^, no peaks are observed in the imino (10–14 ppm) region, indicating that the nucleotide bases are not involved in H-bonds. The appearance of broad signals in the presence of Mg^2+^ confirms the presence of H-bonded nucleotide bases, although the majority of these signals appear over 11 ppm, suggesting the formation of Watson–Crick type base pairs instead of quadruplex structure (Figure S5a).

**Figure 5:**
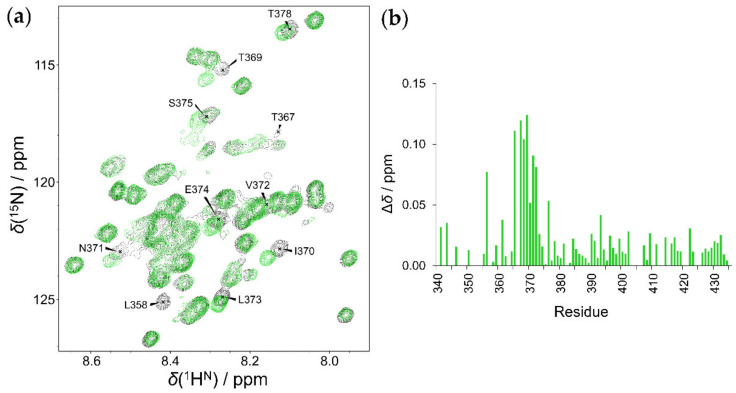
Interaction of HOTAIR_140_ with the EZH2 loop: (**a**) ^1^H-^15^N HSQC of 50 µM wild-type EZH2 in assay buffer (black), and the same protein next to 50 µM HOTAIR_140_ (green). (**b**) Chemical shift perturbation of HOTAIR_140_ on the EZH2 loop.

The interaction and determination of the binding sites on the EZH2 loop was monitored by chemical shift mapping in the absence and presence of Mg^2+^. In both cases, the same result is obtained: the addition of RNA causes significant chemical shift perturbations in the Thr367–Ser375 region (Figure 5). Based on these findings, we can conclude that the secondary structures formed in the HOTAIR_140_ do not have a key role in this interaction. Other regions of the EZH2 loop were not perturbed, and the overall nature of the protein remained highly disordered. The same phenomenon was observed for the T345D mutant (Figure S5b,c). These results point out that the mutated Thr345 and its neighboring residues do not take part in the interaction of the EZH2 loop.

## 3. Discussion

To date, the 96-residue-long loop region of the EZH2 has not been characterized in detail, as its flexible nature prevented its crystallization. The abundance of disorder-promoting prolines (10%) and charged residues (21% negatively and 17% positively charged) together with the depletion of aromatic (1%) and hydrophobic residues (13%) as well as cysteines (1%) suggests that this protein segment lacks a stable 3D fold [29,45]. Indeed, all tested structure prediction methods proposed a highly disordered, random coil-like behavior [21,22,23,24] (Figure 1c). By our detailed NMR spectroscopic investigation, an atomic-level characterization of this EZH2 loop is given.

Regarding the applied experimental set-up, we showed that, while the ^1^H^N^-detected approach fails to deliver satisfactory results at physiological conditions, ^1^H^α^-detected experiments, with further enhanced sensitivity and resolution, can represent a way of reaching the peak assignment in aqueous solutions with 5–10% D_2_O, at 310 K and pH = 7.0. This finding is of general validity for any isotopically labeled IDP.

Analysis of the determined chemical shifts experimentally proved the highly disordered behavior of the EZH2 loop. An α-helical tendency is observed in the Ser408–Ser412 region, and the high disorder is maintained over a large temperature range. This lack of transient structural elements is relatively uncommon even amongst IDPs. EZH2 maintains its highly disordered state even when bound to RNA, and the nascent helical region does not play any role in the interaction. As a counterexample, the disordered p53 transactivation domain possesses nascent helical regions, which undergo a disorder-to-order transition upon binding, while other regions remain fuzzy in the complex [46].

The appearance of *cis*-proline minors was also investigated. For most of the 10 proline residues, we found that the amount of minor *cis*-Pro peaks follows the empirical regularities established from the statistical analysis [30]. Four proline residues (347, 350, 365, and 422) are expected to possess a *cis* conformation lower than 5% due to the presence of positively charged residues in the proline neighborhood at (*i* − 3 and *i* ± 1 positions relative to Pro); indeed, these minors could not be detected. Six proline residues (346, 359, 368, 417, 426, and 427) should give rise to over 5% *cis* conformer, and these do not contain positively charged amino acids at *i* + 3 and *i* ± 1 positions, but are abundant in polar and negatively charged residues in the proline neighborhood. Note that aromatic residues, that can increase *cis*-proline ratio, are not present in the proline proximity in the EZH2 loop. Amongst these six expected *cis* conformers, five were detected with such a ratio, while the minor form of Pro427 could not be detected.

Regarding the phosphomimetic T345D mutant, results revealed that the overall disordered nature of the EZH2 loop is not affected, and that only local changes occur. The neighboring Pro346 is mostly affected, especially its minor *cis* conformer. The significant downfield shift of the *cis*-Pro346 ^1^H^α^ signal indicates a more pronounced turn-like behavior, suggesting that interactions of EZH2 may be affected or regulated via this phenomenon.

Investigation of the EZH2 loop–HOTAIR_140_ interaction shows that, upon binding, EZH2 remains highly unstructured, thus suggesting that a fuzzy complex is formed. Fuzzy interactions are typical to IDP complexes and involve varying levels of flexibility within the bound structures [47]. The binding motif identified here by chemical shift mapping is a nine amino acid-long sequence at Thr367–Ser375. This unique sequence contains various hydrophilic (Ser, Thr, Asn), hydrophobic (Val, Leu, Ile), and a negatively charged (Glu) residue; however, it is devoid of known RNA recognition motifs [48] such as RG repeats, serine-rich motifs, or K/R patches, which are abundant in positively charged amino acids. As the interaction is weak and its specificity is low, it is likely that this binding motif cooperates with other RNA-binding sites throughout the whole EZH2 protein [49]. We also point out that—at least in the case of the isolated loop region—the G-quadruplex structure of the RNA is not essential for the recognition. Furthermore, the negative charge at the 345th position does not affect the interaction significantly.

**Figure 6:**
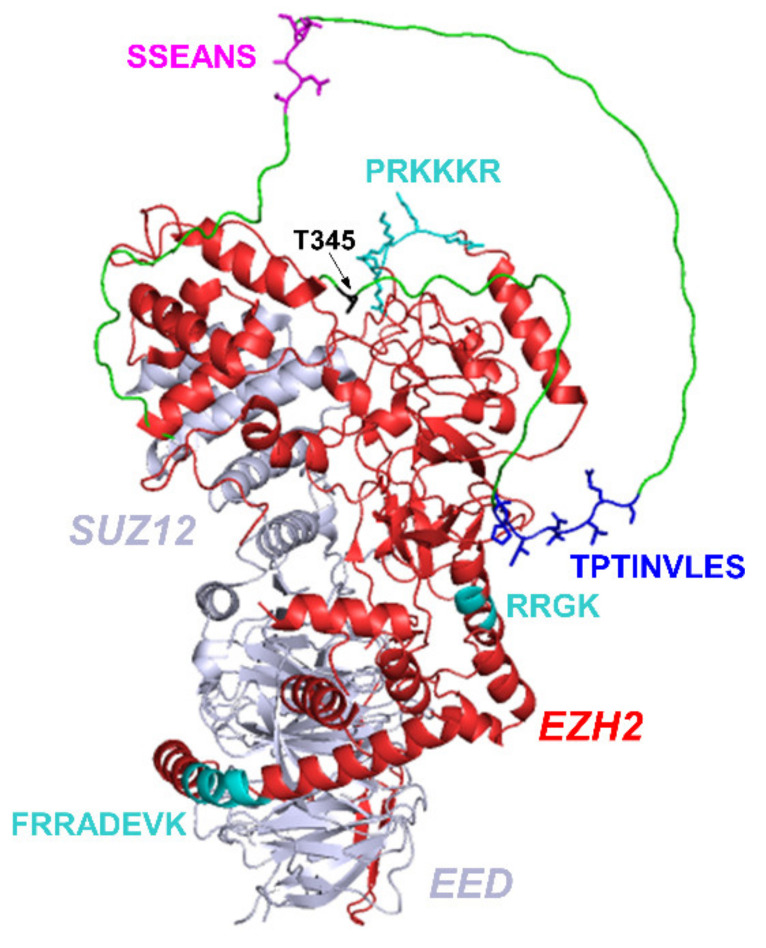
RNA-binding motifs and other important regions identified in the present work. PRC2 crystal structure (PDB: 5LS6) includes SUZ12, EED (grey), and EZH2 (red) [6]; disordered regions were added from the AlphaFold structure (see Figure 1b). The studied EZH2 loop region is highlighted with green. RNA-binding regions are marked with cyan (earlier work [18]) and with blue (this study), with the amino acid sequences added. The EZH2 loop’s nascent helical region and its sequence is shown with magenta. Position of phosphomimetic mutation (T345) is marked with black.

Taken together, our results suggest a rather non-specific, fuzzy interaction of the EZH2 loop with HOTAIR_140_ lncRNA. Given that the investigated region of EZH2 is localized on the surface of the PRC2 complex, it is ideally placed to serve as a first recognition point during the RNA-binding process. RNA molecules anchored to this region then have the opportunity to form high-affinity interactions with other, more difficult-to-access binding surfaces on the PRC2 (Figure 6). It is also important to note that the earlier identified [18] binding surfaces recognize G-quadruplex structures, whereas the loop studied here does not show a clear preference for this structure. This further supports the suggestion that it provides an initial, non-specific binding surface for RNAs possibly acting together with the other, G-quadruplex-specific disordered binding motif localized in its proximity (Figure 6). Based on our structural observations, how phosphorylation of Thr345 increases the affinity towards RNA it is not readily apparent, as it is located far from the RNA-binding region, although it may be possible that the more turn-like tendency of the *cis*-proline minor that appears upon phosphorylation has a somewhat more favorable shape that is complementarity with the partner RNAs.

## 4. Materials and Methods

### 4.1. EZH2 Loop Expression and Purification

The same methods of protein overexpression and purification were used for both protein constructs, EZH2^wt^ loop and mutant EZH2^T345D^ loop. DNA sequence coding for wild-type protein was purchased from Eurofins Genomics (Ebersberg, Germany) and subcloned into pET22b (+) cloning vector. The T345D mutant was generated using site directed mutagenesis method (forward primer: ACGCATCAAAGACCCGCCT, reverse primer: TCAGCATCCATGGCCATC). The pET-22b (+) vector containing the appropriate construct was transformed into competent *E. coli* BL21 * (DE3) pLysS cells and grown in LB medium containing 0.05 mg/mL carbenicillin overnight at 37 °C with shaking at 180 rpm. After inoculation with the starter cell culture into fresh LB medium containing 0.05 mg/mL carbenicillin, the cells were grown to OD_600_ = 0.8 and transferred one hour prior to induction to M9 minimal medium, complemented with ^15^N-labeled ammonium chloride and ^13^C-labeled glucose (Eurisotop, Saint-Aubin, France). Induction was done for 4 h at 37 °C by 0.5 mM IPTG, then cells were pelleted by centrifugation (4000 rpm, 20 min, 4 °C) and incubated at room temperature for 30 min in lysis buffer (20 mM Tris, 200 mM NaCl, 20 mM imidazole, 0.1% Triton X-100, 1 mg/mL Lysozyme, 50 U/mL Nuclease (Pierce™ Universal Nuclease for Cell Lysis, Thermo Fisher Scientific, Waltham, MA USA) pH 7.5 and EDTA-free SIGMAFAST Protease Inhibitor Cocktail Tablets, Merck KGaA, Darmstadt, Germany) with vigorous shaking. After sonication, the cell debris was removed by centrifugation (12,100 rpm, 40 min, 4 °C). The supernatant was filtered through 0.2 μm nitrocellulose filter then purified over HisTrap HP (Merck KGaA, Darmstadt, Germany) column on an AKTA Explorer system using a gradient elution of two buffers (Buffer A: 20 mM imidazole, 200 mM NaCl, 20 mM Tris. pH 7.5. Buffer B: 1 M imidazole, 200 mM NaCl, 20 mM Tris, pH 7.5). Representative purification results are shown on Figure S6. Elution fractions containing sufficiently pure proteins were boiled for 10 min then pelleted by centrifugation (14,000 rpm, 20 min, 4 °C). The supernatant was transferred to distilled water via buffer exchange on a HiPrep 26/10 desalting column (GE Healthcare, Chicago, IL, USA). The elution fractions were lyophilized and stored at −20 °C until usage.

### 4.2. RNA Transcription and Purification

HOTAIR_140_ (1–140 nt) DNA sequence cloned into pEX-A128 vector was purchased from Eurofins Genomics (Ebersberg, Germany). After 2 h digestion with EcoRV restriction enzyme at 37 °C, the gel-purified, linearized DNA template was used to synthesize RNA by in vitro transcription carried out with New England BioLabs HiScribe™ T7 Quick High Yield RNA Synthesis Kit (Ipswich, MA, USA). After transcription, remaining DNA template was eliminated with DNaseI treatment. RNA sample purification was carried out using Macherey-Nagel NucleoSpin^®^ RNA Clean-up XS Kit (Düren, Germany). The quality and intactness of the purified transcription product was analysed by native and formaldehyde agarose gel electrophoresis. Final RNA concentration was determined using Implen NanoPhotometer™ N60 Spectrophotometer (Münich, Germany). Purified RNA was stored at −80 °C until usage in the presence of RNAINH-RO Roche Protector RNase Inhibitor (20 U). Before usage the RNA sample was refolded by incubation at 75 °C for 5 min and then allowed to cool to room temperature.

### 4.3. NMR Measurements

For NMR measurements, the lyophilized protein samples were dissolved in PBS buffer (pH = 7.0) containing 10 mM TCEP and 3 mM NaN_3_, then 5% D_2_O and 1% DSS (Eurisotop, Saint-Aubin, France) were added. Final concentration of the protein was (0.5–1.0) mM in each NMR sample. For protein–RNA-binding studies, the ^15^N labelled wild type or the T345D mutant EZH2 loop was dissolved in assay buffer (see Scheme S1) containing 5% sterile filtered D_2_O. Final concentration of the protein was 50 μM.

All NMR measurements were performed on a Bruker Avance III 700 MHz spectrometer (700.05 MHz for ^1^H, 176.03 MHz for ^13^C, 70.94 MHz for ^15^N) equipped with a Prodigy TCI H&F-C/N-D probehead (Ettingen, Germany). ^1^H^N^-detected experiments for backbone assignment at 278 K were: 2D ^1^H-^15^N correlations (Fast-HSQC [50] and SOFAST-HMQC [51]), BEST-type triple resonance experiments [52,53] (HNCA, HN(CO)CA, HNCACB, HN(CO)CACB, HNCO, HN(CA)CO), CcccoNH [54], and TOCSY-HSQC [55]. Performed ^1^H^α^-detected experiments: 2D SHACA-HSQC [32], 3D HCAN [34], HCA(CO)N [34,56,57], and HCA(N)CO [58] were conducted at 278 K and 310 K. For proline conformation detection, the proline side chain selective Pro-(H)CGCBCAHA experiment was performed [30]. Temperature dependence of SHACA-HSQC and SOFAST-HMQC were measured in the range of (278–310) K.

## Data Availability

Chemical shift assignments were deposited in BMRB with the following entry numbers: 51420 (EZH2^wt^) and 51421 (EZH2^T345D^).

## Supplementary Materials

The following supporting information can be downloaded at: https://www.mdpi.com/article/10.3390/ijms23116150/s1.
