# Supplementary material for: The Disordered EZH2 Loop: Atomic Level Characterization by 1HN- and 1Hα-Detected NMR Approaches, Interaction with the Long Noncoding HOTAIR RNA
Source: Int J Mol Sci. 2022 May 30;23(11):6150. doi: 10.3390/ijms23116150 (PMC9181245; doi:10.3390/ijms23116150)
Supplement: Supplementary file 1 [file ijms-23-06150-s001.zip › ijms-1737587-supplementary.pdf]

Supplementary Information for

The Disordered EZH2 Loop: Atomic Level Characterization by $^{1}H^{N}$- and $^{1}H^{\alpha}$-Detected NMR Approaches, Interaction with the Long Noncoding HOTAIR RNA

Csenge Lilla Szabó[1,2], Beáta Szabó[3], Fanni Sebák[1], Wolfgang Bermel[4], Ágnes Tantos[3]*, Andrea Bodor[1]*

[1] Analytical and BioNMR Laboratory, Institute of Chemistry, Eötvös Loránd University, Pázmány Péter sétány 1/A, 1117 Budapest, Hungary

[2] Hevesy György PhD School of Chemistry, Eötvös Loránd University, Pázmány Péter sétány 1/A, 1117 Budapest, Hungary

[3] Institute of Enzymology, Hungarian Academy of Sciences, Magyar tudósok körútja 2, 1117 Budapest, Hungary

[4] Bruker BioSpin GmbH, Rudolf-Plank Str. 23, 76275 Ettlingen, Germany

* Correspondence: tantos.agnes@ttk.hu (A.T.); andrea.bodor@ttk.elte.hu (A.B.)

Table of content

Scheme S1. Composition of assay buffer. 2

Figure S1. Glycine region of SHACA-HSQC[1] for EZH2. 3

Figure S2. Improvement of HCAN to eliminate signal loss at 310 K. 4

Figure S3. Structural propensities of EZH2 loop. 6

Figure S4. Identification of proline minors. 7

Figure S5. Interaction of $EZH2^{T345D}$ with $HOTAIR_{140}$. 8

Figure S6. Coomassie Blue stained SDS-PAGE analysis of the purified recombinant His-tagged proteins. 9

Scheme S1. Composition of assay buffer.

1. PBS buffer was treated with DEPC for sterilization.
2. Theoretical solubility of Mg^{2+} in $c_{phosphate}$ = 10 mM, pH = 7.0 PBS buffer (K_{sp} = $[Mg^{2+}]^3 \cdot [PO_4^{3-}]^2 = 1.04 \cdot 10^{-24}$ $(mol/dm^3)^5$): 1.56 mM. The solubility of Mg^{2+} in PBS is highly sensitive to phosphate concentration and pH, so solubility has to be checked. In our case, 2.5 mM $MgCl_2$ was added to the PBS buffer, and neither precipitation nor opalescence of the solution was observed.
3. Other components: 0.05 % NP40 and 1 mM DTT were added.

Figure S1. Glycine region of SHACA-HSQC [1] for EZH2. For Gly352 and Gly356 geminal couplings are clearly visible.

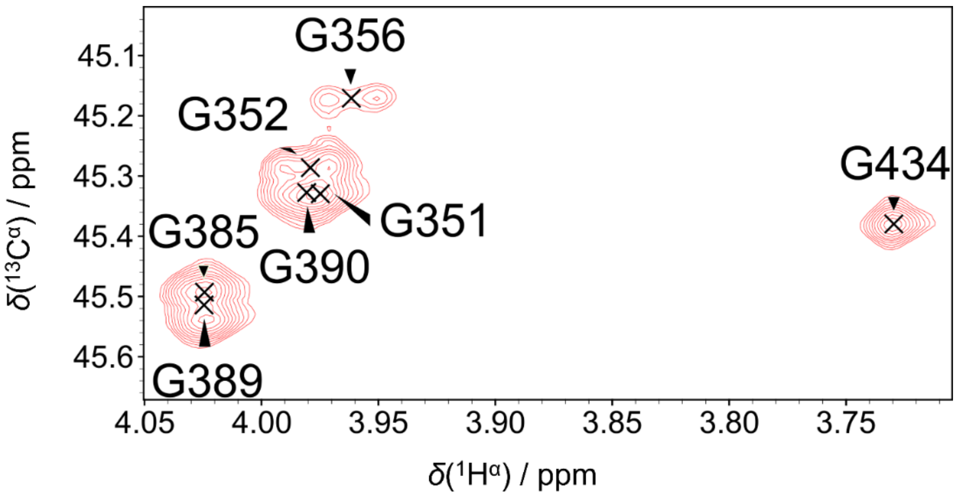

Figure S2. Improvement of HCAN to eliminate signal loss at 310 K. (a) Improvements introduced to the classical HCAN pulse program (hcangp3d) [2]. Note, corresponding delay times were also adjusted. (b) Improvements highlighted on the original Bruker hcangp3d pulse-sequence. (c) Strips of original Bruker HCAN pulse sequence. (d) Strips of the improved HCAN.

(**a**)

Improvements introduced to the classical HCAN pulse program (hcangp3d). Note, corresponding delay times were also adjusted.

1. Weak presaturation (pl32)
2. A pair of low power 90° pulses
   p26 ph1
   p26 ph2
   (at pl19, ph1=0, ph2=1)
3. Purge block
   20u pl10:f1
   (p17 ph1)
   (p17*2 ph2)
   4u
   p30:gp4
   d16 pl1:f1
   (p1 ph1)
   4u
   p31:gp4
   d16
   4u pl19:f1
   Note, in the original HCAN pulse sequence a p30:gp4 spoil gradient was already applied.
4. Gradients in back-INEPT: a pair of p19: gp5 (600us), and a pair of p16:gp2 (1ms)
5. BASEREX detection scheme

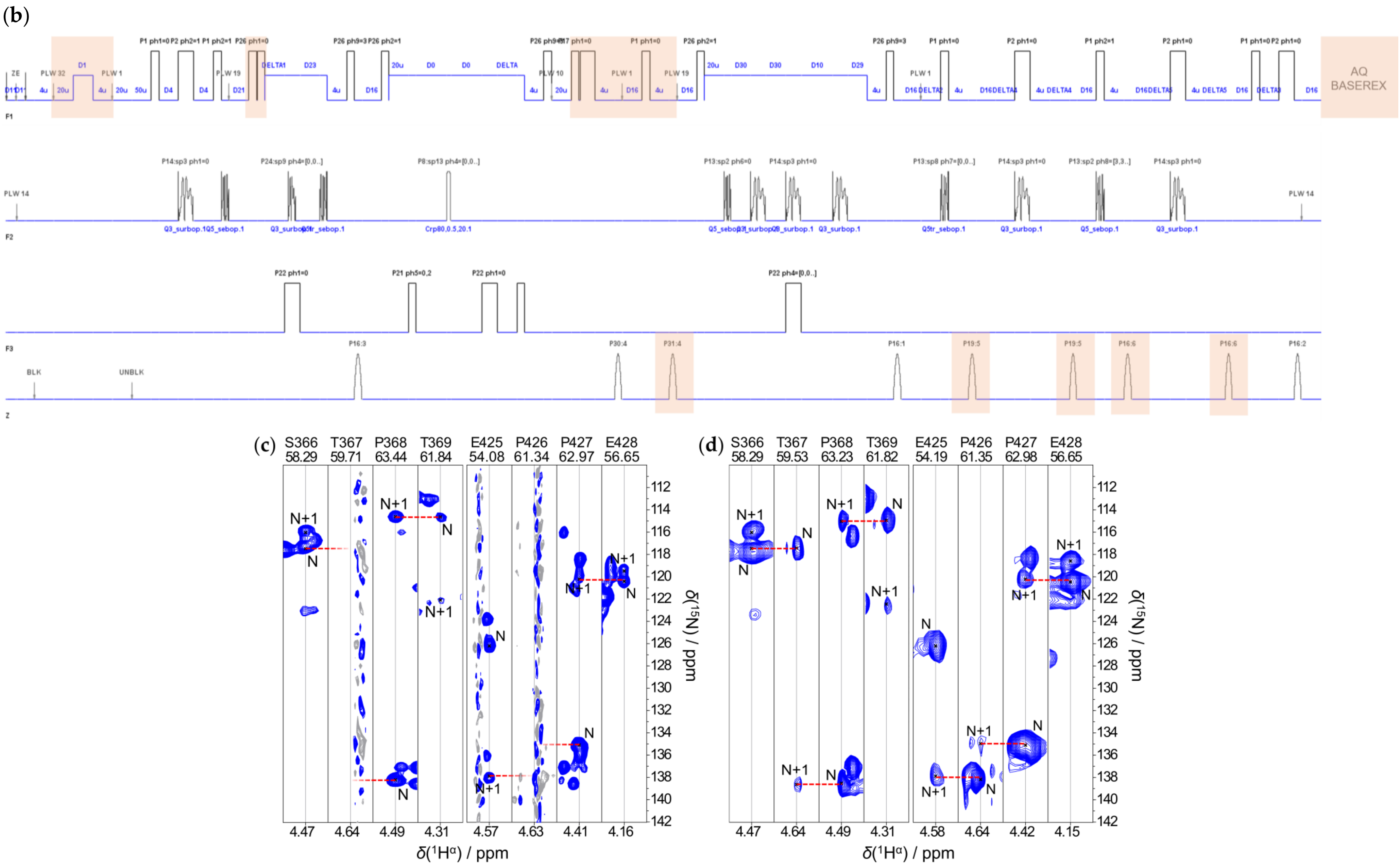
(b)
AQ
BASEREX
F1
F2
F3
Z
BLK
UNBLK
PLW 14
(c)
S366
58.29
T367
59.71
P368
63.44
T369
61.84
E425
54.08
P426
61.34
P427
62.97
E428
56.65
N
N+1
δ(¹Hα) / ppm
δ(¹⁵N) / ppm
(d)
S366
58.29
T367
59.53
P368
63.23
T369
61.82
E425
54.19
P426
61.35
P427
62.98
E428
56.65
δ(¹Hα) / ppm
δ(¹⁵N) / ppm

Figure S3. Structural propensities of EZH2 loop. (a) pH dependence of SCS values at 278 K. (b) Temperature dependence of SCS values at pH = 7.2. Random coil chemical shifts were calculated with the predictor of Kjaergaard et al. [3,4] (c) SSP values of EZH2 at 310 K, pH = 7.0 (red), at 278 K, pH = 7.0 (blue) and at 278 K, pH = 5.1 (black). (d) Temperature coefficients of amide protons (pH = 7.0), determined from data collected at 278 K – 310 K range.

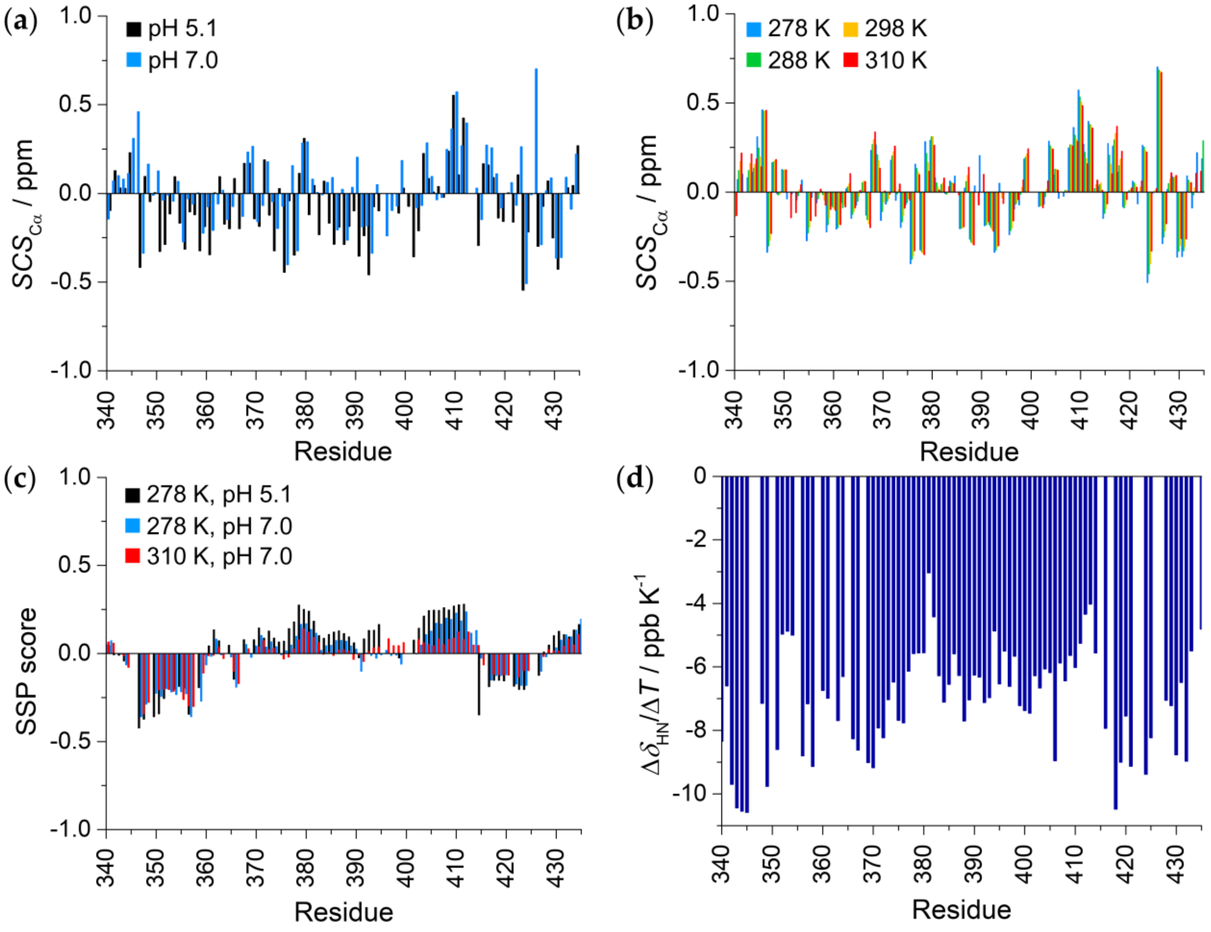

Figure S4. Identification of proline minors. (a) Proline region of SHACA-HSQC [1] (EZH2^{wt}, 310 K). Minor signals are indicated with the respectivce lowercase letters. (b) Strips of the 3D Pro_CGCBCAHA [5], indicating the proline $^{13}C^{\beta}$ and $^{13}C^{\gamma}$ chemical shifts of Pro417, Pro346 and their *cis* conformers.

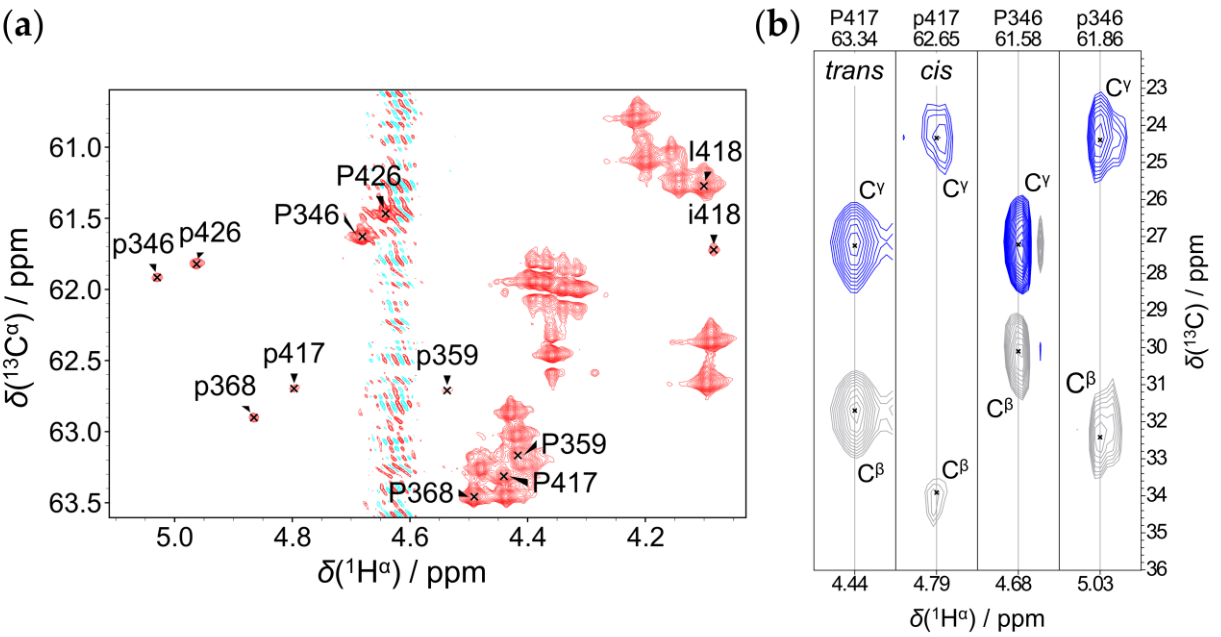

Figure S5. Interaction of EZH2^{T345D} with HOTAIR$_{140}$. (a) Imino region of HOTAIR$_{140}$ 1D ^{1}H spectrum. (b) ^{1}H-^{15}N HSQC of 50 μM EZH2^{T345D} in assay buffer (black) and the same protein next to 50 μM HOTAIR$_{140}$ RNA (red). (c) Chemical shift perturbations caused by HOTAIR$_{140}$ on EZH2^{T345D}.

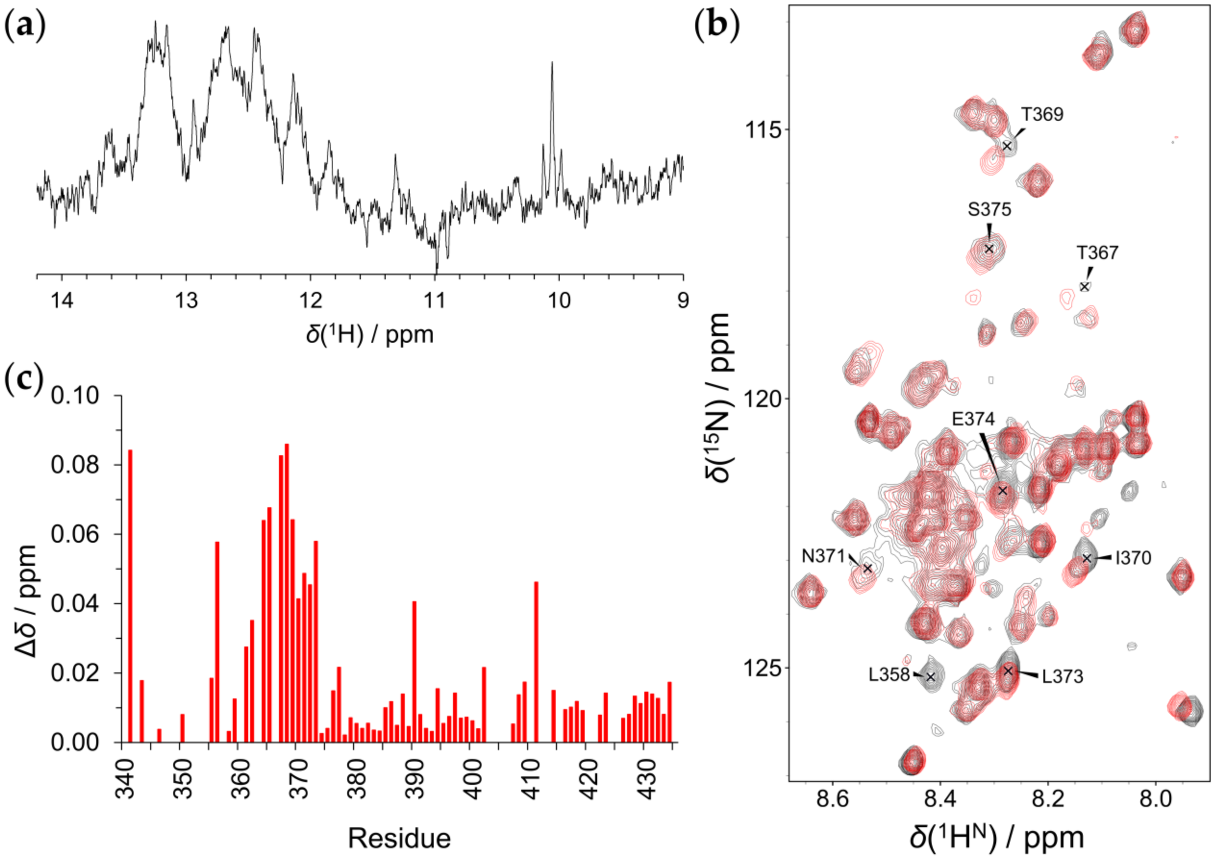

Figure S6. Coomassie Blue stained SDS-PAGE analysis of the purified recombinant His-tagged proteins.

Lane 1. PageRuler™ Plus Prestained Protein Ladder (Thermo Scientific™, 26619), Lane 2. 10 mg ^{15}N, ^{13}C EZH2^{wt} loop, Lane 3. 10 mg ^{15}N EZH2^{T345D} loop

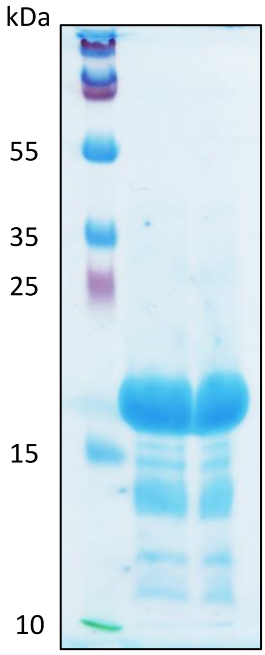

References:

1. Bodor, A.; Haller, J.D.; Bouguechtouli, C.; Theillet, F.X.; Nyitray, L.; Luy, B. Power of Pure Shift HαCα Correlations: A Way to Characterize Biomolecules under Physiological Conditions. *Anal. Chem.* **2020**, *92*, 12423-12428, doi:10.1021/acs.analchem.0c02182.
2. Kanelis, V.; Donaldson, L.; Muhandiram, D.R.; Rotin, D.; Forman-Kay, J.D.; Kay, L.E. Sequential assignment of proline-rich regions in proteins: Application to modular binding domain complexes. *J. Biomol. NMR* **2000**, *16*, 253-259, doi:10.1023/A:1008355012528.
3. Kjaergaard, M.; Brander, S.; Poulsen, F.M. Random coil chemical shift for intrinsically disordered proteins: effects of temperature and pH. *J. Biomol. NMR* **2011**, *49*, 139-149, doi:10.1007/s10858-011-9472-x.
4. Kjaergaard, M.; Poulsen, F.M. Sequence correction of random coil chemical shifts: correlation between neighbor correction factors and changes in the Ramachandran distribution. *J. Biomol. NMR* **2011**, *50*, 157-165, doi:10.1007/s10858-011-9508-2.
5. Sebák, F.; Ecsédi, P.; Bermel, W.; Luy, B.; Nyitray, L.; Bodor, A. Selective (1) H(α) NMR Methods Reveal Functionally Relevant Proline cis/trans Isomers in Intrinsically Disordered Proteins: Characterization of Minor Forms, Effects of Phosphorylation, and Occurrence in Proteome. *Angew. Chem. Int. Ed. Engl.* **2022**, *61*, e202108361, doi:10.1002/anie.202108361.
